# Radiographic Predictors of Shunt Dependency in Intracranial Hemorrhage With Intraventricular Extension

**DOI:** 10.7759/cureus.28409

**Published:** 2022-08-25

**Authors:** James Brazdzionis, Paras Savla, Stacey Podkovik, Ira Bowen, Emilio C Tayag, Michael Schiraldi, Dan E Miulli

**Affiliations:** 1 Neurosurgery, Riverside University Health System Medical Center, Moreno Valley, USA; 2 Neurology and Neurosurgery, Desert Regional Medical Center, Palm Springs, USA; 3 Neurosurgery, Redlands Community Hospital, Redlands, USA; 4 Neurosurgery, Desert Regional Medical Center, Palm Springs, USA; 5 Neurosurgery, Arrowhead Regional Medical Center, Colton, USA

**Keywords:** ventriculoperitoneal shunt, risk, radiographic, intraventricular hemorrhage, intracranial hemorrhage

## Abstract

Background

Intracranial hemorrhage (ICH) may be complicated by intraventricular hemorrhage (IVH) and hydrocephalus, which can require the placement of a ventriculoperitoneal shunt (VPS). ICH and IVH risk scores using radiographic and clinical characteristics have been developed but utilization for assessment of future need for VPS placement is limited.

Methods

This is a single-institution retrospective review for patients with primary ICH with IVH from 2018-2020. Initial CTs and charts were analyzed to determine ICH, IVH, LeRoux and Graeb scores, Evans’ index, ICH and IVH volumes, and comorbidities. Outcomes including Glasgow coma scale (GCS), National Institute of Health Stroke Scale (NIHSS), length of stay, and shunt placement were evaluated with bivariate correlations, t-tests, chi-squared tests, and receiver operating characteristic (ROC) curves (p=0.05).

Results

A total of 130 patients were included of which 102 underwent full treatment beyond hospital day one. VPS placement was significantly associated with longer length of stay (p<0.001), discharge NIHSS (p=0.001), arrival Evans’ index (p<0.001), IVH (p=0.033), LeRoux (p=0.049), but not comorbidities, ICH score, or admission GCS. When treated beyond hospital day one, Evans’ index (p<0.001), IVH volume (p=0.029), Graeb (p=0.0029), IVH (p=0.004), Slice (p=0.015), and Leroux scores (p=0.006) were associated with shunt placement of which an Evans’ index of 0.31 or greater had highest sensitivity and specificity (area under the ROC curve (AUC) 0.81, sensitivity 81%, specificity 0.76).

Conclusions

The higher the Evans’ index, Graeb, IVH, Slice, and LeRoux scores on admission, the higher the risk of shunt dependency in patients undergoing full treatment beyond hospital day one. Admission imaging scores significantly predict the development of shunt dependence and may be considered in treatment.

## Introduction

Hemorrhagic stroke or intracranial hemorrhage (ICH) is a devastating disease that affects approximately 78,000 people in the United States per year with a prevalence of 232-270/100,000 [1,2]. Mortality rates at one year are as high as 50%, and are typically higher in hemorrhagic strokes as compared to ischemic strokes; however, both may result in significant morbidity [1-4]. Treatment is geared towards promoting an environment to reduce secondary injury, reducing risk factors for recurrent hemorrhage in the future, and building rehabilitative efforts over the long term. Hemorrhagic stroke has been investigated, and scales such as the ICH score have been developed to predict mortality and better educate patients and families regarding the severity of the disease and potential outcomes [5]. One factor within the ICH score is intraventricular hemorrhage (IVH). IVH may be present in 30-45% of ICH patients and is therefore not an uncommon clinical factor [6]. This prognostic factor has been controversial within the literature in terms of its functionality of prediction of mortality, as each intraventricular hemorrhage may have varying characteristics including volume of hemorrhage, distribution of hemorrhage, and presence of hydrocephalus, which as a binary score does not account for these differences [4,6,7]. To address these variables, different scores including the IVH score, Graeb score, LeRoux score, and Slice score have been developed [6,8-10]. Each score has been determined to correlate with morbidity and mortality in patients with IVH in differing settings [6-10].

A known complication related to IVH is hydrocephalus. Hydrocephalus is a risk factor for increased morbidity and mortality [4,7]. To combat hydrocephalus, patients with signs of elevated intracranial pressure (ICP) on arrival due to their hemorrhage or those determined to be clinically at risk of development of hydrocephalus may have an intracranial pressure monitor or external ventricular drain (EVD) placed. Additionally, in patients with ICH with IVH, EVDs may be placed to deliver thrombolytic therapy within the ventricle in selected patients [11,12]. Once an EVD is placed, ICP and cerebrospinal fluid (CSF) outputs are monitored and can be titrated to treat the hydrocephalus, keeping ICPs below 20mmHg to prevent secondary brain injury. Furthermore, ICP trends evaluate the effectiveness of CSF diversion through the EVD and provide an indirect measurement of the patient’s intrinsic CSF clearance mechanisms. These CSF clearance mechanisms may be adversely affected by the IVH and patients may become dependent on a shunt to maintain CSF homeostasis. In these patients, typically ICP remains elevated despite gradual weaning of an EVD, with concomitant increases in CSF output. In order to continue to treat these patients and their resultant hydrocephalus, the EVD typically is converted to a ventriculoperitoneal shunt (VPS) for a more permanent diversion.

Determining which patients may require a VPS is a nuanced task and generally takes place as a patient fails a weaning trial of the EVD. However, current literature lacks evidence-based guidelines on typical EVD weans including the rate of wean, the timing of wean, or predictability of long-term failure after wean [13]. Therefore, most neurosurgeons or neurosurgical centers have developed their own practices of weaning an EVD and determining of need for VPS. Our center’s protocol utilizes mmHg as a height with an EVD typically initially placed at 6 mmHg. After a patient is stabilized medically and has stable ICPs, the EVD is weaned with height changes every 24 hours. The height of the EVD typically progresses as follows 6 mmHg, 10 mmHg, 15 mmHg, 20 mmHg. After reaching 20 mmHg for 24 hours, the EVD is clamped for 24 hours with a CT scan taken the morning after clamping. If ventricular size remains normal on this post-clamp CT scan and ICP remains within normal limits during the 24 hours of clamp trial, the EVD is removed. If the patient fails the clamp trial, whether through the development of hydrocephalus or with elevations of ICP during the trial, the EVD is opened and the wean may be restarted the next day or the patient will be designated as shunt dependent and will be scheduled for a VPS placement.

In other circumstances, the surgeon elects to proceed directly to shunt placement. However, VPS placement has risks and complications including high failure rate and infection, which have been well documented in adult and pediatric populations [14,15]. Therefore, when possible, many neurosurgeons prefer to wean the EVD and avoid the intrinsic surgical risks and post-placement risks that come with VPS placement. However, these risks are balanced by prolonged intensive care unit length of stays, cost of treatment, and other risks associated with prolonged hospital stay that occur due to a prolonged EVD wean. Further risks of long-term CSF diversion with an EVD include the risk for infection and risks associated with immobility including deep vein thrombosis, deconditioning, and nosocomial infection [16,17].

Previous studies have attempted to investigate risk factors associated with a need for VPS in patients with ICH with IVH; however, results were mixed and varied [18,19]. These studies included only patients with initial EVD placement and not all patients with ICH with IVH, and had differing weaning parameters than our own. As IVH and ICH are a continuum and not all patients may have an EVD placed initially, we wished to include all patients with an ICH and IVH to better approximate the population as a whole as ICH with IVH in our analysis. We retrospectively investigated risk factors within our patient population that can be obtained on arrival including radiographic characteristics, comorbidities, and clinical status that are available early within hospitalization to predict the need for VPS placement.

## Materials and methods

After approval of the Institutional Review Board, Arrowhead Regional Medical Center, Colton, California, United States (approval number 21-01), this retrospective review was carried out in Arrowhead Regional Medical Center, Colton, California, United States, utilizing an internal neurosurgical database that includes all patients treated by the neurosurgery service at our center. A waiver of informed consent was granted by our institutional review board due to the retrospective nature of this trial. The retrospective review investigated all patients admitted from January 2018 through December 2020. Patients were included for analysis if they were over the age of 18 years, were diagnosed with an ICH, and had IVH on admission. Exclusion criteria included nonspontaneous ICH (vascular abnormalities such as arteriovenous malformation or aneurysm), hemorrhagic mass, hemorrhagic conversion of ischemic stroke, and patients under the age of 18.

Demographic characteristics including age, sex, and race were collected. Comorbidities of atrial fibrillation, anticoagulation use, antiplatelet use, diabetes, hypertension, hyperlipidemia, chronic kidney disease, end-stage renal disease, hypothyroidism, coronary artery disease, alcohol use, cirrhosis, and previous stroke were collected. ICH score was evaluated through a retrospective chart review of the patient’s record with ICH score on admission (or when hemorrhage occurred while the patient was admitted for an alternative reason, at the time of hemorrhage) was collected. IVH, Graeb score, LeRoux score, and Slice score was calculated through evaluation by a neurosurgery resident’s review of the patient’s admission CT scan. Volume of IVH was calculated using verified conversion of IVH score to volume [8]. ICH volume was obtained through a review of medical records or when not calculated in neurosurgical charting was calculated from a review of the initial CT scan using the standard ABC/2 method. Length of stay, duration of EVD placement, mortality, withdrawal of care, National Institute of Health (NIH) stroke scale on discharge (documented by nursing staff on discharge), modified Rankin score (documented by nursing staff on discharge), discharge location, admission Glasgow Coma Scale (GCS), discharge GCS, surgical procedures, and VPS placement were all evaluated. The Evans’ index was calculated using an established cut-off of 0.3 for hydrocephalus on arrival through a retrospective review of the initial CT scan by a neurosurgery resident [20].

Statistical analysis was performed using open-source GNU PSPP statistical software (GNU Project, Boston, Massachusetts, United States). Correlations were evaluated using two-sided Pearson correlation coefficients. Categorical variables were assessed using Chi-square tests. Multivariate logistic regression was utilized to investigate relationships between variables. Univariate analysis was conducted on variables with statistical significance with analysis using odds ratios (OR) with 95% confidence intervals. A receiver operating characteristic (ROC) curve was computed using statistical software. All p-values utilized an alpha level of 0.05. Demographics data were compared with t-tests and Chi-squared analysis.

## Results

A total of 130 patients who had spontaneous ICH with IVH from January 1, 2018, through December 31, 2020, were included in the analysis, of which 49 patients underwent EVD placement. Of those that underwent EVD placement, 16 failed EVD wean and required shunting. A total of 39 were treated with intrathecal tissue plasminogen activator (tPA) per our center’s protocol, which included 2mg tPA every 12 hours starting 24 hours after stable hemorrhage on repeat CT scan. The average patient age was 60.59 and 71 patients were male. When assessing for differences in the shunt group with the non-shunt group, there were no significant differences in sex (p=0.355), age (p=0.180), and race (p=0.427). Length of stay was significantly different between both groups (p<0.001) with an average length of stays of 14.34 days overall, 11.38 days in the no shunt group, and 35.44 days in the shunted group. The overall median ICH score was 2.72 in all patients, and the ICH score was not associated with shunting (p=0.446). The average length of time an EVD remained in place was 10 ± 10.69 days. Overall mortality was 54 patients (41.5%). When excluding patients with the withdrawal of care within hospital days zero and one, the mortality decreased to 28/102 patients or 28.5%. The average length of stay in patients without withdrawal of care on days zero and one was 17.99 days overall, 14.74 days in the non-shunted group, and 35.44 days in the shunted group (p<0.001). Demographics and patient characteristics for all patients are tabulated in Table 1 and demographics and patient characteristics for patients with whom care was not withdrawn on hospital days zero and one in Table 2.

**Table 1:** Demographics and associated characteristics in all patients with spontaneous intracranial hemorrhage with intraventricular extension Demographics data were compared with t-tests and Chi-squared analysis ICH: intracranial hemorrhage

	Shunt (N=16)	No shunt (N=114)	P-value
Age (years)	55.94	61.25	P=0.180
Sex			P=0.355
Male	7	64	
Female	9	50	
Race			P=0.427
Asian	0	4	
African American	4	14	
White	4	42	
Hispanic	8	54	
Length of Stay	35.44	11.38	P<0.001
ICH score	2.50	2.75	P=0.446

**Table 2:** Demographics and associated characteristics in patients receiving care beyond hospital day one Demographics data were compared with t-tests and Chi-squared analysis ICH: intracranial hemorrhage

	Shunt (N=16)	No shunt (N=86)	P-value
Age (years)	55.94	61.09	P=0.197
Sex			P=0.479
Male	7	46	
Female	9	40	
Race			P=0.392
Asian	0	4	
African American	4	10	
White	4	31	
Hispanic	8	41	
Length of Stay	35.44	14.74	P<0.001
ICH score	2.50	2.34	P=0.588

In investigating clinical factors and imaging characteristics related to ventriculoperitoneal shunt placement, multivariate analysis identified that shunting was significantly correlated with multiple admission characteristics. The factors used were determined using factors from other previously created scores. Associations included Evans’ index, admission IVH score, and admission LeRoux score. Further factors associated with shunting included significant effects on length of stay, GCS at discharge, EVD placement, length of EVD placement, usage of tissue plasminogen activator (tPA), NIH score, and a negative correlation with mortality. These are tabulated in Table 3.

**Table 3:** Significant relationships in all patients with spontaneous ICH with intraventricular extension associated with shunt placement GCS: Glasscow coma scale; IVH: intraventricular hemorrhage; EVD: external ventricular drain; tPA: tissue plasminogen activator; NIH: National Institutes of Health; ICH: intracranial hemorrhage

Category	Pearson Coefficient	P-value
Length of Stay	0.484	<0.001
Evans’ Index	0.310	<0.001
GCS at Discharge	0.186	0.035
IVH Score on Admission	0.188	0.033
Leroux Score	0.173	0.049
Placement of an EVD	0.482	<0.001
Length of EVD Placement	0.505	<0.001
Usage of intrathecal tPA	0.466	<0.001
Mortality	-0.316	<0.001
NIH Score	0.397	0.001

When the analysis excluded patients with mortality related to withdrawal of care on days zero and one, to better assess patients receiving full treatment of their underlying injury, additional characteristics with significant effects were noted. In these patients, shunt placement was significantly associated with length of stay, Evans’ index, Slice score, Graeb Score, IVH score, IVH volume, LeRoux score, placement of an EVD, length of EVD placement, usage of tPA, discharge NIHSS, and was negatively associated with mortality. However, when controlling for the presence of an EVD, usage of tPA was no longer significantly associated with shunt rate. ICH score and ICH volume were not associated with the eventual need for shunt placement. Usage of anticoagulation, antithrombotics, and comorbidities of diabetes, hypertension, hyperlipidemia, chronic kidney disease, end-stage renal disease, coronary artery disease, hypothyroidism, alcohol use, cirrhosis, and previous stroke had no significant correlation with shunting. Modified Rankin score at discharge and discharge GCS was not associated with shunting. Furthermore, GCS on arrival, age, and sex were also not significant factors in the eventual need for VPS placement in this population. Pearson correlation coefficients for significant factors associated with shunting are seen in Table 4.

**Table 4:** Significant relationships in patients receiving care beyond hospital day one with spontaneous ICH with intraventricular extension associated with shunt placement IVH: intraventricular hemorrhage; EVD: external ventricular drain; NIHSS: National Institutes of Health Stroke Scale; tPA: tissue plasminogen activator; ICH: intracranial hemorrhage

Category	Pearson Coefficient	P-value
Length of Stay	0.452	<0.001
Evans’ Index	0.383	<0.001
Slice Score	0.239	0.015
Graeb Score	0.256	0.009
IVH Score	0.284	0.004
IVH Volume	0.216	0.029
Leroux Score	0.271	0.006
Placement of an EVD	0.467	<0.001
Length of EVD Placement	0.493	<0.001
Usage of intrathecal tPA	0.429	<0.001
Mortality	-0.265	0.007
NIHSS on Discharge	0.397	0.001

To investigate cut-off values for predictability of these individual factors in patients who did not have care withdrawn on days zero or one, analysis using ROC curves was utilized. When using an Evans’ index cut-off of 0.31, the sensitivity and specificity to predict shunting were 0.81 and 0.76, respectively, with an area under the curve (AUC) of 0.81 (p<0.001, CI 0.73-0.88). A Slice score greater than 9.5 was 75% sensitive and 65% specific for shunting (AUC= 0.61, p=0.020 CI 0.58-0.79). If the Graeb score was greater than 5.5 it was 81% sensitive but only 52% specific for the need for shunt placement (AUC=0.70, p<0.009, CI 0.59-0.82). An IVH score of greater than 10.5 was significantly associated with the need for VPS placement (AUC=0.73, p=0.003, CI 0.64-0.82) with a sensitivity of 94% but was only 49% specific. When using an IVH score cut-off of greater than 11.5, it was 75% sensitive and 68% specific for VPS placement. IVH volume cut-offs of greater than 10.02 and 8.97 were 75% sensitive and 68% specific and 94% sensitive but 49% specific, respectively (AUC=0.73, p=0.003, CI 0.64-0.82). LeRoux scores demonstrated a p-value of 0.006 and CI of 0.62-0.81. When using a LeRoux score cutoff of greater than 9.5, it was 75% sensitive and 60% specific in predicting the need for VPS in patients with ICH with IVH who did not have mortality due to withdrawal of care on hospital days zero and one. The overall AUC for LeRoux score was 0.72.

## Discussion

Although shunt dependency in the setting of ICH with IVH is a well-known phenomenon, there is limited data in the literature predicting the need for placement of a VPS. Our data set identifies that patients with higher Evans’ indices, IVH scores, and Leroux scores on initial evaluation had significantly higher rates of VPS placement. These correlations were significant even when including patients following early withdrawal of care on hospital days zero and one within the “no shunt” group, despite the likelihood that the underlying hemorrhage was severe enough that shunting may have been required should the patient have survived the initial injury.

When investigating patients that underwent the full spectrum of treatment and excluding those with care withdrawn prior to hospital day two, additional radiographic factors were found to have significant correlations with shunt placement yielding significant cut-offs that may be considered in clinical practice. These factors included the Evans’ index, Slice score, Graeb score, IVH score, LeRoux score, and IVH volume calculated by IVH score. As described in the results section, each one of these characteristics was powered enough to develop cut-off values for shunt placement. The Evans’ index had a significant cut-off of 0.31 in which a score of 0.31 or greater was 81% sensitive and 76% specific in determining the need for shunt placement in this retrospective cohort that survives beyond hospital day one, regardless of any other additional imaging characteristic. Thus, the neurosurgeon may have a higher degree of suspicion for shunt dependence in patients with spontaneous ICH with IVH that have elevated Evans’ indices of 0.31 or greater and may consider that the patient is likely to have the EVD successfully weaned out if the initial Evans’ index is less than 0.31. The other radiographic characteristics of Slice score, Graeb Score, IVH score, IVH volume, and LeRoux Score also demonstrated significant associations and cut-offs with higher sensitivity than specificity. When using cut-offs of 9.5, 5.5, 10.5, 10.02, and 9.5 for Slice score, Graeb score, IVH score, IVH volume, and LeRoux Score, respectively, there was much greater sensitivity than specificity for shunt placement. Therefore, these values are far more likely to identify patients that would go on to develop shunt-dependent hydrocephalus than those that would not. Thus, these criteria may be considered in practice as cut-offs that may be utilized to more expeditiously identify patients that may go on to require shunting. Therefore, if patients have a Slice score lower than 9.5, a Graeb score less than 5.5, IVH score less than 10.5, IVH volume calculated by IVH score less than 10.02, and LeRoux score less than 9.5, the index of suspicion for the eventual need for VPS placement may be significantly lower and these patients may more than likely go on to have their EVD weaned and removed. Overall predictability with the highest AUC was found in Evans’ index (0.81) followed by IVH score and IVH volume (both 0.73), LeRoux score (0.72), Graeb score (0.70) and Slice score (0.61). Thus, the highest predictability was found in Evans’ index with Slice score having the weakest predictability using cutoffs.

Outcomes were also analyzed and patients that went on to undergo shunting had less mortality regardless of whether withdrawal of care on days zero and one were excluded; however, they had higher NIH scores on discharge. These higher NIH scores may demonstrate the higher degrees of injury in patients that develop shunt-dependent hydrocephalus compared to patients that do not. Modified Rankin score and GCS at discharge were not found to be statistically significant between shunted versus non-shunted patients. Clinical characteristics including initial GCS, ICH score, and comorbidities on admission were not associated risk factors for the development of shunt-dependent hydrocephalus in our subset. Therefore, radiographic risk factors may better prognosticate risk for shunting in this patient population than standard clinical examination on admission.

On initial analysis, tPA was also found to be a factor related to VPS placement; however, when patients who did not undergo EVD placement were excluded, it was no longer a significant factor. In our practice, each patient who does not have a vascular lesion or other contraindication to tPA receives intrathecal tPA as an adjunctive measure to promote clearance of the IVH. Therefore, this significant correlation initially evaluated was likely just a factor of more patients receiving tPA within the subset shunt group than a true effect. The benefits of tPA in regards to the reduction of hemorrhage have been investigated through the CLEAR trial, but despite improvements in hemorrhage volume, these effects were not correlated with the eventual need for shunt placement [12,18]. Additionally, within the CLEAR III results, they were not able to identify initial imaging findings that correlated with the need for a shunt [18]. Therefore, our data add to the literature and identify significant radiographic characteristics that may influence the overall treatment of spontaneous ICH with IVH patients. 

Additional studies have demonstrated thalamic hemorrhage as a common underlying variable correlated with ventriculoperitoneal shunt placement [21,22]. Further characteristics associated with shunting varied by study but some selected variables previously associated with the need for VPS included GCS score, significant lateral ventricular blood, elevations in intracranial pressure, presence of hydrocephalus, IVH score, bicaudate index, fourth ventricular blood volume, and ratio of blood in the third and fourth ventricles [19,21,22]. Each of these studies had differing weaning parameters using cm H2O in Kuo et al. and Miller et al. with no described wean protocol in Zacharia et al. [19,21,22]. These differing characteristics in weaning may affect the determination of shunt dependence. Our center utilizes mmHg as a standard setting with weaning occurring stepwise with increases from an initial setting of 6mmHg to 10mmHg, 15 mmHg, and 20 mmHg followed by a 24-hour clamp trial in 24-hour increments when determined safe by the operating neurosurgeon. In comparison to the previous literature, our center’s data agreed that measurements of hydrocephalus (in our case Evans’ index) were significantly correlated to the development of shunt-dependent hydrocephalus [19]. However, our study investigated additional scoring systems beyond the IVH score previously explored by Kuo et al. and found correlations with LeRoux score and Graeb score not seen in Zacharia et al. [19,22]. Our study is the first to investigate Slice score as a predictor of shunt dependence in spontaneous ICH.

Intra-hospital care in patients with ICH with IVH, especially for patients undergoing CSF diversion with an EVD, has a pronounced cost. Patients with an EVD are monitored in an ICU setting while the EVD remains in place. ICU stays can be costly and while a patient is undergoing an EVD wean, typically they have largely been medically stabilized except for the need for removal of EVD or placement of a shunt. These associated weans and prolonged treatment requirements may lead to increased hospital lengths of stay. Unsurprisingly, this was demonstrated in our population in which patients who underwent shunt placement had significantly longer length of stay than those that did not (p<0.001) with an average length of stay of 35.44 days in the shunted group compared to 11.38 days in all patients with ICH with IVH that did not undergo shunting. Additionally, post VPS placement, if not already performed and the patient has suffered a significant enough injury to necessitate, a gastrostomy tube may be required to be placed for long-term feeding. Some surgeons elect to wait till after VPS placement for gastrostomy placement due to concerns over seeding of CSF with abdominal flora [23]. This “waiting period” may additionally result in a prolonged hospitalization with increased length of stay and cost. Other concerns about increased length of stay include risks of hospital-acquired infections such as pneumonia, deep venous thrombosis, delays in initiating rehabilitative efforts, and other factors. If patients are found to be at higher risk for VPS placement due to their arrival Evans’ index, IVH score, IVH volume, LeRoux score, Graeb score, or Slice score, a higher index of suspicion may be considered, and it may be considered to place a VPS sooner due to increased risks of failure of EVD wean.

Limitations of this study include the retrospective nature of this trial. Additionally, there was no long-term follow-up data available for study within this population. Our study did not investigate the methodology of characteristics that may affect shunt placement such as comparing differing weaning parameters. Future studies may wish to investigate EVD weaning practices to establish further evidence-based protocols as well as prospectively evaluate patients with ICH with IVH to determine additional factors related to shunting and corroborate the information in this trial as well as those by Kuo et al., Miller et al., and Zacharia et al. [19,21,22].

## Conclusions

We report that overall Evans’ index, IVH score, IVH volume, LeRoux score, Graeb score, and Slice score on admission and CT scan were significantly related to the development of shunt-dependent hydrocephalus in patients with spontaneous ICH with IVH. Evans’ index had the highest degree of predictive value while Slice score was the weakest predictor. Using Evans’ index of 0.31 or greater had 81% sensitivity and 76% specificity. As ICH with IVH is a complex disorder with varying outcomes and high degrees of morbidity and mortality, understanding factors that can be associated with procedures that may result in future hospitalizations, increased care requirements, and prolonged hospitalization are critical to promote optimal patient care. Surgeons may consider utilizing these factors in discussion with patients and families to provide education as well as utilizing these factors within their own pre-surgical planning processes to optimize patient care; specifically with consideration of repeated EVD wean in patients at lower risk for shunt placement versus consideration of earlier shunt placement in patients who are failing or failed wean with higher risk for development of shunt-dependent hydrocephalus.
